# Spectroscopic studies of sequence-dependent conformational transitions in asymmetric G/C rich double-stranded DNA

**DOI:** 10.1007/s00249-025-01767-7

**Published:** 2025-06-12

**Authors:** Petra Školáková, Iva Kejnovská, Daniel Renčiuk

**Affiliations:** Institute of Biophysics of the Czech Academy of Sciences, Královopolská 135, 612 65 Brno, Czech Republic

**Keywords:** Circular dichroism spectroscopy, DNA, Guanine quadruplex, Cytosine i-motif, Conformation equilibrium

## Abstract

Nucleic acids, molecules essential for all life, can adopt many alternative structures besides the well-known right-handed double helix, some of which have been reported to exist and function in vivo. One of the most appropriate methods for structural studies of nucleic acids is circular dichroism spectroscopy, utilizing structure-induced chirality due to the asymmetric winding of absorbing nucleobases. Using electronic CD and absorption spectroscopies in combination with melting experiments, we analyzed a conformational equilibrium between DNA double helix and two alternative conformations of nucleic acids, cytosine i-motifs and guanine quadruplexes, as a function of the primary structure of model G/C-rich sequences, containing blocks of G and C runs in particular DNA strands. This paper is a part of special issue dedicated to 70th anniversary of the Biophysical Institute of the Czech Academy of Sciences, where circular dichroism spectroscopy of nucleic acids has been used successfully and impactfully for many years.

## Introduction

Nucleic acids are key molecules of all living organisms, storing their genetic information, allowing its expression into organism phenotype and playing a key role in the adaptation to environmental changes. DNA is mainly considered a structurally and functionally uniform stable storage of genetic information, while RNA with its structural variability covers several different roles mostly in the expression of genetic information. The local conformational variability of nucleic acids, especially DNA, represented by various alternative structures might serve as a fine-tuning regulatory mechanism of nuclear processes including replication, transcription or DNA repair (Wang and Vasquez 2023).

DNA in living systems is usually in the form of a right-handed double helix stabilized by several factors: hydrogen bonds in base pairs, stacking of bases, hydration and compensation of negative phosphate groups repulsion by ions (Neidle 1999). The distribution of hydrogen bond donors and acceptors within bases, either natural or induced by base modifications, allows canonical Watson–Crick pairing of bases, but also non-canonical base pairs, which might lead to the formation of alternative DNA secondary structures. These are then the result of an optimal combination of primary sequence, allowing base pairing and base stacking, and environmental conditions providing proper hydration and stabilizing ions. Recently the most studied alternative nucleic acids structures are probably cytosine i-motifs (iM) (Gehring et al. 1993; Tao et al. 2024), and guanine quadruplexes (G4) (Sen and Gilbert 1988), forming in nucleic acids rich in cytosine and guanine, respectively, when these are located in several consecutive Gn or Cn tracts (further denoted here as asymmetric G/C-rich). Both these alternative structures were reported in cells using specific antibodies: BG4 for G4 (Biffi et al. 2013) and iMab for iM (Zeraati et al. 2018). The human genome is full of sequences able to form G4 (Chambers et al. 2015) and iM (Peña Martinez et al. 2024), but the occurrence of iM in vivo is still under debate (Dzatko et al. 2018; Víšková et al. 2024; Zeraati et al. 2018). These motifs are frequent in gene promoters and enhancers or heterochromatic regions such as telomeres or centromeres. In complementary double-stranded DNA, such as eukaryotic nuclear DNA, the motifs for potential iM and G4 formation thus lie in the same regions but in the opposite strands.

**Fig. 1:**
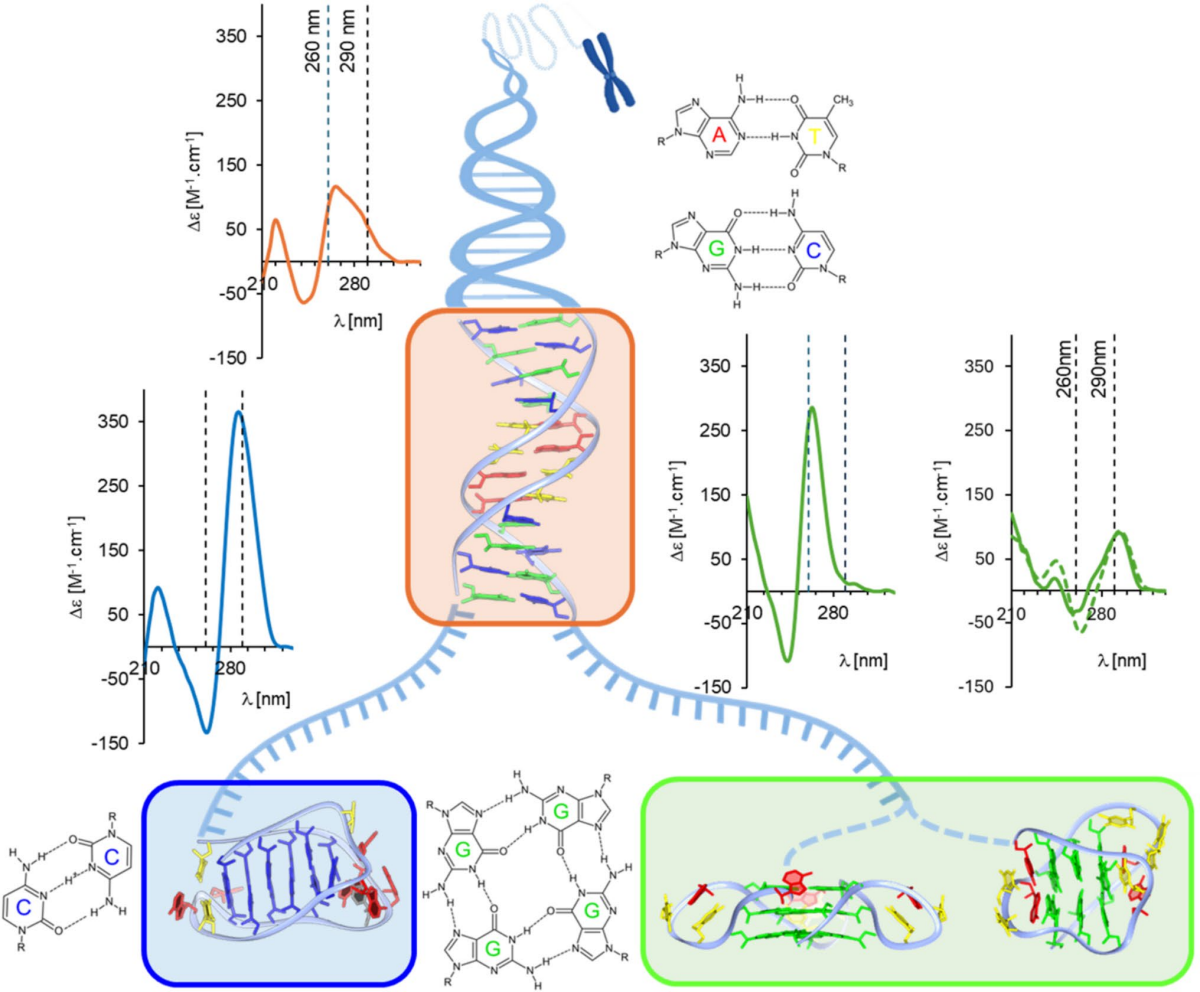
Schematic representation of canonical B-DNA double helix (orange) and alternative DNA structures, which might form on complementary G- and C-rich strands: cytosine i-motif (blue), based on presence of C:C^+^ base pairs, and two types of guanine quadruplex (green), namely parallel and antiparallel one, both based on presence of guanine tetrads. CD spectra of the structures in buffer with K^+^ (1xIC) are shown in respective colors. Dashed spectrum of antiparallel G4 was observed in a buffer with Na^+^ instead of K^+^. Central DNA scheme was created in https://BioRender.com

The primary structural factor of cytosine iM is a hemi-protonated cytosine: cytosine (C:C^+^) pair (Fig. 1, bottom left). The protonation of free cytidine at N3 position has pKa around 4.2, thus the formation of iM should be limited to acidic pH conditions. However, the incorporation of cytidine into oligonucleotide capable to form iM shifts the pKa to neutral or even slightly alkaline values (Wright et al. 2017). The thermodynamic stability of iM then quite well correlates with pH and also with the number of consecutive C:C^+^ pairs (Dvorakova et al. 2018) starting from 3 pairs at pH 5 for stable iM at 0 °C (Ghezzo et al. 2023). The overall structure of the iM core comprises two antiparallelly intercalated duplexes, each composed from two C:C^+^ paired parallelly oriented strands, forming a four-stranded block-shaped structure with two narrow and two wide grooves (Fig. 1, bottom left). Such composition does not allow efficient base stacking, but the iM might be stabilized by hydration, although the effect of crowding/dehydrating agents on iM stability is not clear; both stabilizing (Saxena et al. 2017) and neutral role were reported (Jamroskovic et al. 2022). Also the effect of various ions and their concentration on the iM stability is rather complex; ions might stabilize iM by shielding negative phosphates but also might decrease the stability of C:C^+^ pairs (Kim and Hong 2023). iM is also stabilized by incorporation into double-stranded DNA (Rodriguez et al. 2023), which could potentially extend its lifetime in the genome. The formation of iM proceeds through the kinetic partitioning mechanism, where a number of metastable structures differing in the presence of nonpaired C in the iM cores, form in parallel and the interconversion between these structures or transition to more thermodynamically optimal ones is limited (Školáková et al. 2023).

The stabilization factors of G4 differ from those of iM: G4 is based on the pairing of guanines through two hydrogen bonds between Watson–Crick surface of one G and the Hoogsteen surface of the other in a planar square-shaped fashion containing four guanines and eight hydrogen bonds, a so-called guanine tetrad (Fig. 1, bottom right). Already free GMP molecules form tetrads that strongly stack over each other (Gellert et al. 1962). Stacked tetrads arrangement leads to the formation of a central cavity in and between them, associated with a negative charge due to guanine O6 carbonyl groups, which must be compensated by proper cation to stabilize G4. G4 is thus extremely sensitive to cation type and concentration, while the best stabilizers are potassium ones that perfectly fit into G4 central cavity between two neighboring tetrads. The whole quadruplex then comprises several guanine tetrads stacked over each other and forming core and loop regions interconnecting the guanine tracts that form tetrads (Fig. 1, bottom right). Due to the combined effect of hydrogen bonds, guanine stacking and ion coordination, which all are related to the formation of guanine tetrads, the number of tetrads is the key determinant of quadruplex stability. Several topologies of G4 were described, depending on the mutual orientation of the bound guanine tracts: parallel, antiparallel or hybrid, each associated with different loop types. The topologies differ also in the conformation of glycosidic angles in guanosines; parallel quadruplex comprises all *anti* G, whereas antiparallel ones involve an organized matrix of both *syn* and *anti*-guanosine conformations (Neidle and Balasubramanian 2006). Not all combinations of G4 topology and primary sequence are allowed. To span proper guanosine tracts in an antiparallel topology, at least a two-nucleotide loop is necessary. In contrast, single-nucleotide loops are always propeller (Hazel et al. 2004), limiting the G4 topology to parallel or hybrid. In specific cases, also the sequence of loops might significantly influence the G4 stability and even conformation (Guédin et al. 2009; Guedin et al. 2008). G4s are sensitive to hydration level with precisely coordinated water molecules in grooves (Li et al. 2021). Interestingly, dehydration induced by crowding or dehydrating agents might stabilize the G4 and even change its conformation towards more parallel ones (Renciuk et al. 2009).

The studies of the equilibria between the asymmetric G/C-rich DNA double helix and alternative structures, such as iM and G4, have been reported for more than 20 years. The first reports focused on human telomeric DNA, specifically variants of the G_3_(TTAG_3_)_3_ fragment, studied through spectroscopic methods (Li et al. 2002) and NMR (Phan and Mergny 2002). These studies showed a key effect of pH on the equilibrium, with more acidic pH shifting it towards alternative structures, mainly due to the stabilization of the iM in the C-rich strand. Single-molecule studies of long telomeric fragments suggest that stable alternative structures remain only if both iM and G4 are stabilized, i.e., under high K^+^ concentration and low pH (Zhang et al. 2020). More detailed analysis of various DNA fragments, including telomere ones, by CD and fluorescence methods, coupled with subsequent multivariate data analysis (Jaumot et al. 2006) revealed the coexistence of G4 and double helix, while the equilibrium depended on the G4 conformation and stability, which might be influenced by loop regions. The effect of loop length on the equilibrium of the double helix versus alternative structure has also been described in more detail (Kumar et al. 2008), showing that increasing loop length allows better interaction with complementary C-rich strand and shifts the equilibrium towards double-stranded DNA. Subsequently, the GC-rich region of the c-kit gene promoter was analyzed in a similar manner (Bucek et al. 2009). Analogous studies have shown that regions overlapping the G4 or iM core shift the equilibrium towards the double helix (Arora et al. 2009), but double-helical regions adjacent to G4 can be destabilized and usually require the presence of a several-nucleotide long connection region (König et al. 2013). It has been shown that the coexistence of iM and G4 on complementary chains within the double helix is penalized and these structures are mutually exclusive (Dhakal et al. 2012; Kendrick and Hurley 2010). The same has subsequently been generalized to other G4-prone fragments, such as the telomeric repeat and promoter regions of the hTERT and bcl-2 genes (Cui et al. 2016). A study of the human telomeric fragments, the thrombin-binding aptamer quadruplex and G4 in the c-myc gene promoter using fluorescence resonance energy transfer, subsequently showed that the kinetic parameters of the structures at equilibrium correlate with the thermodynamic stability of these structures (Mendoza et al. 2015), and that equilibrium can be shifted by appropriate ligands stabilizing the respective structures (Tran et al. 2021). Appropriate substitutions, for example, by 1′,2′-dideoxyribose, disrupting Watson–Crick pairing of complementary chains can also lead to a shift in equilibrium, as has been shown for the N-myc gene promoter fragment (Trajkovski and Plavec 2020). Most recent studies focused, for example, on the conformational equilibrium in the hexanucleotide repeats GGGGCC associated with amyotrophic lateral sclerosis/frontotemporal dementia (Diggins et al. 2024).

One of the most suitable methods for structural studies of nucleic acids is circular dichroism spectroscopy. CD spectroscopy historically played an important role in structural studies of nucleic acids: it forecasted the existence of left-handed DNA double helix, Z-DNA (Pohl and Jovin 1972) or a hybrid type of guanine quadruplex (Vorlickova et al. 2005a). Due to its simplicity and reliability, it is a standard method for conformation checks, not only for nucleic acids but also for proteins. Significant contribution to the application of CD spectroscopy to nucleic acids structure determination can be attributed to the Institute of Biophysics of the Czech Academy of Sciences, namely the research team led by Michaela Vorlíčková (Kejnovska et al. 2019; Kypr et al. 2009; Vorlickova et al. 2005b). The CD spectrum of the standard right-handed B form double helix is plain with a small positive band around 275 nm and a negative one at 245 nm. However, depending on the primary structure, the DNA structure and accordingly its CD spectrum can change distinctly. It is also the case of highly asymmetric sequences such as poly dG:poly dC double helix (Kypr et al. 2009). The common spectral feature of the asymmetric dG:dC rich sequence is a positive CD peak with a maximum around 260 nm and a negative one in the 240 nm region (Fig. 1, upper left). Cytosine i-motif, stabilized by hemi-protonated C:C^+^ base pairs, is characterized by a dominant positive CD peak around 290 nm (Fig. 1, bottom left). iM forms are structurally uniform structures with most of the variability only in loop or overhang parts, and small variability in the position of the first C:C^+^ pair with respect to the 5′ end of the molecule, so called 5′E and 3′E conformation, which is not reflected in the CD spectra. The spectra are quite uniform; however, the height of the positive CD peak reflects the number of C:C^+^ pairs (Školáková et al. 2019). In contrast, guanine quadruplexes potentially formed in the complementary strand are conformationally broad due to the variability of mutual strand orientation and subsequent conformation of guanine glycosidic angles. Their combinations between neighbor guanines lead to significant differences in CD spectra (Fig. 1, bottom right): parallel quadruplexes are characterized by spectra with a high positive peak around 265 nm, whose height correlates with a number of guanine tetrads involved. The antiparallel ones provide spectra with a dominant positive peak around 290 nm and a negative peak around 260 nm. Hybrid-type quadruplexes share spectral signatures of both (Kejnovska et al. 2019; Kypr et al. 2009). The measured CD spectra of complex mixtures represent a weighted average of spectra of individual components, while the balances are their ratios. In our study the mixture might contain a double helix of G- and C-rich strands, these strands are separated and unfolded or folded into iM or various types of G4. In terms of CD spectral characteristics, some conformations overlap, for example, both iM and antiparallel G4 provide positive CD peaks around 290 nm, but the short-wavelength parts of their spectra differ. From the comparison of the CD spectra of the mixture of the two complementary strands and the average of the CD spectra of the two strands separated, we can determine whether a change in secondary structure occurred, for example, if the double helix was formed.

In the study, we employed the CD spectroscopy for a brief analysis of model G- and C-rich sequences and their mixtures to reveal additional aspects of the conformational equilibria, namely the effect of a number of tetrads/C:C^+^ pairs and loop sequence and length, both in combination with neutral and mildly acidic pH. CD spectroscopy was combined with a simple analysis of the thermodynamics of the structures, obtained by absorption spectroscopy. The main goal of this work is to highlight CD spectroscopy as a fast, inexpensive and extremely sensitive tool for studies of conformation and conformational transitions of nucleic acids.

## Methods

### Oligonucleotides

Synthetic oligonucleotides were purchased from Merck/Sigma-Aldrich (Haverhill, UK). Oligonucleotides were desalted and lyophilized by the provider. Lyophilized oligonucleotides were dissolved in 1 mM Na-phosphate buffer and 0.3 mM EDTA, pH 7 to a 10 mM nucleoside concentration. The precise concentration of oligonucleotides was determined from their absorption at 260 nm in 1 mM Na-phosphate buffer, pH 7 at 90 °C, using calculated molar absorption coefficients (ε) as described earlier (Kejnovska et al. 2019). All sequences used, their labels and ε are summarized in Table 1. Heteroduplexes were prepared by mixing equimolar amounts of cytosine- and guanine-rich strands.

**Table 1:** Sequences of DNA used in the study together with their labels, lengths in nucleotides, molar absorption coefficients in [M^−1^ cm^−1^] and Tm and Tren (in °C) in respective pH values

Label	Oligonucleotide	nt	ε	pH 7.5		pH 6		
				T_m_	T_ren_	T_m_	T_ren_	
C3T	TTACCCTCCCTCCCTCCCATT	21	8300	–	–	11.1		10.7
G3A	AATGGGAGGGAGGGAGGGTAA	21	11,300	81.7	82.1	83.6		85.8
dx		42	9800	61.6	62.8	60.5		61.3
C3T2	TTACCCTTCCCTTCCCTTCCCATT	24	8300	–	–	27.6		27.9
G3A2	AATGGGAAGGGAAGGGAAGGGTAA	24	11,400	34.5	42.9	45.2		47.9
dx		48	9800	66.6	67.3	64.3		63.1
C3T3	TTACCCTTTCCCTTTCCCTTTCCCATT	27	8300	–	–	32		32
G3A3	AATGGGAAAGGGAAAGGGAAAGGGTAA	27	11,500	43.6	39.5	43.7		44
dx		54	9900	66.8	67.7	62.8		66.3
C3T4	TTACCCTTTTCCCTTTTCCCTTTTCCCATT	30	8300	–	–	34.1		31.8
G3A4	AATGGGAAAAGGGAAAAGGGAAAAGGGTAA	30	11,500	10.7	12.3	–		–
dx		60	9900	66.6	67	62.3		64.2
C4T	TTACCCCTCCCCTCCCCTCCCCATT	25	8100	–	–	37.3		36.5
G4A	AATGGGGAGGGGAGGGGAGGGGTAA	25	11,200	75.4	72.1	77.5		74.5
dx		50	9700	70	71.4	62.9		69.2
C4T2	TTACCCCTTCCCCTTCCCCTTCCCCATT	28	8200	–	–	41.8		41.8
G4A2	AATGGGGAAGGGGAAGGGGAAGGGGTAA	28	11,300	74	74.9	77.5		77.8
dx		56	9700	68.6	71	64.1		69.8
C4T3	TTACCCCTTTCCCCTTTCCCCTTTCCCCATT	31	8200	–		44.2		42.5
G4A3	AATGGGGAAAGGGGAAAGGGGAAAGGGGTAA	31	11,300	85.5	75.7	82.1		82.1
dx		62	9800	69.9	71	65.3		66.4
C4T4	TTACCCCTTTTCCCCTTTTCCCCTTTTCCCCATT	34	8200			46		40.9
G4A4	AATGGGGAAAAGGGGAAAAGGGGAAAAGGGGTAA	34	11,400	60.2	54.3	78.1		65.1
dx		68	9800	68.2	70.5	65.1		66.5
C4A	TTACCCCACCCCACCCCACCCCATT	25	8700	–	–	34.6		33.9
G4T	AATGGGGTGGGGTGGGGTGGGGTAA	25	10,600	86.1	90.9	77.1		76.4
dx		50	9600	69.1	71.6	71.1		71.9
C4A2	TTACCCCAACCCCAACCCCAACCCCATT	28	9000	–	–	34.4		36.2
G4T2	AATGGGGTTGGGGTTGGGGTTGGGGTAA	28	10,400	84	82.4	75.7		76
dx		56	9700	70.2	71.7	70.8		71.3
C4A3	TTACCCCAAACCCCAAACCCCAAACCCCATT	31	9300	–	–	42.5		41.3
G4T3	AATGGGGTTTGGGGTTTGGGGTTTGGGGTAA	31	10,200	86.7	85.8	86.3		80
dx		62	9800	70.7	70	70.3		71.1
C4A4	TTACCCCAAAACCCCAAAACCCCAAAACCCCATT	34	9500	–	–	44.1		37.8
G4T4	AATGGGGTTTTGGGGTTTTGGGGTTTTGGGGTAA	34	10,100	79	75.7	67.6		66.2
dx		68	9800	73.9	74.5	71.3		72.5

### Spectroscopy of circular dichroism

CD measurements were conducted using a J-815 dichrograph (Jasco, Japan) in 1 cm path-length cuvettes (Hellma, Germany). Spectra were collected as the average of four accumulations between 330 and 210 nm with data pitch 0.5 nm and D.I.T. 1 s at 200 nm min^−1^ scan speed. CD signals are expressed as the difference in the molar absorption, Δε, of the left- and right-handed circularly polarized light and the molarity is related to strands. The experimental conditions were changed directly in the cuvettes by adding “intracellular” buffer (IC), to a 1 × concentration, i.e. 25 mM sodium phosphate buffer, 110 mM KCl, 10 mM NaCl, 1 mM MgCl_2_. The pH values were adjusted by adding 2 M HCl and measured using a Mettler Toledo pH meter with an InLab micro pH electrode. The final strand concentrations were corrected for volume increase and were 2–3 μM for each sample in the cuvette. The CD melting experiments were taken within two temperature ramps ranging from 15 to 90 °C and 90 to 15 °C, respectively, in 5 °C steps and the average temperature rate was 0.8 °C min^−1^. Additional details of sample preparation and conditions for individual experiments are listed in respective figure legends.

### Absorption spectroscopy

Absorption spectra and melting experiments were conducted on a Specord 250 plus UV/Vis spectrophotometer (Analytik Jena, Germany). The whole spectra were taken within three temperature ramps ranging from 5 to 95 °C, 95 to 5 °C and again 5 to 95 °C, in 1 °C steps. Including measurement time, the average temperature decrease/increase rate was 0.25 °C min^−1^. Each spectrum was measured between 330 and 230 nm with data pitch 1 nm at a scan rate of 600 nm min^−1^. Denaturation and renaturation curves were monitored by absorbance at 297 nm (for quadruplexes and i-motifs) and 260 nm (for heteroduplexes). The data were normalized using dual baseline correction according to Mergny and Lacroix (2003). The melting temperatures (Tm) and midpoint renaturation temperatures (Tren) are expressed as the temperature where half of the sample is folded.

## Results and discussion

### CD spectroscopy clearly follows complex DNA conformation transitions

**Fig. 2:**
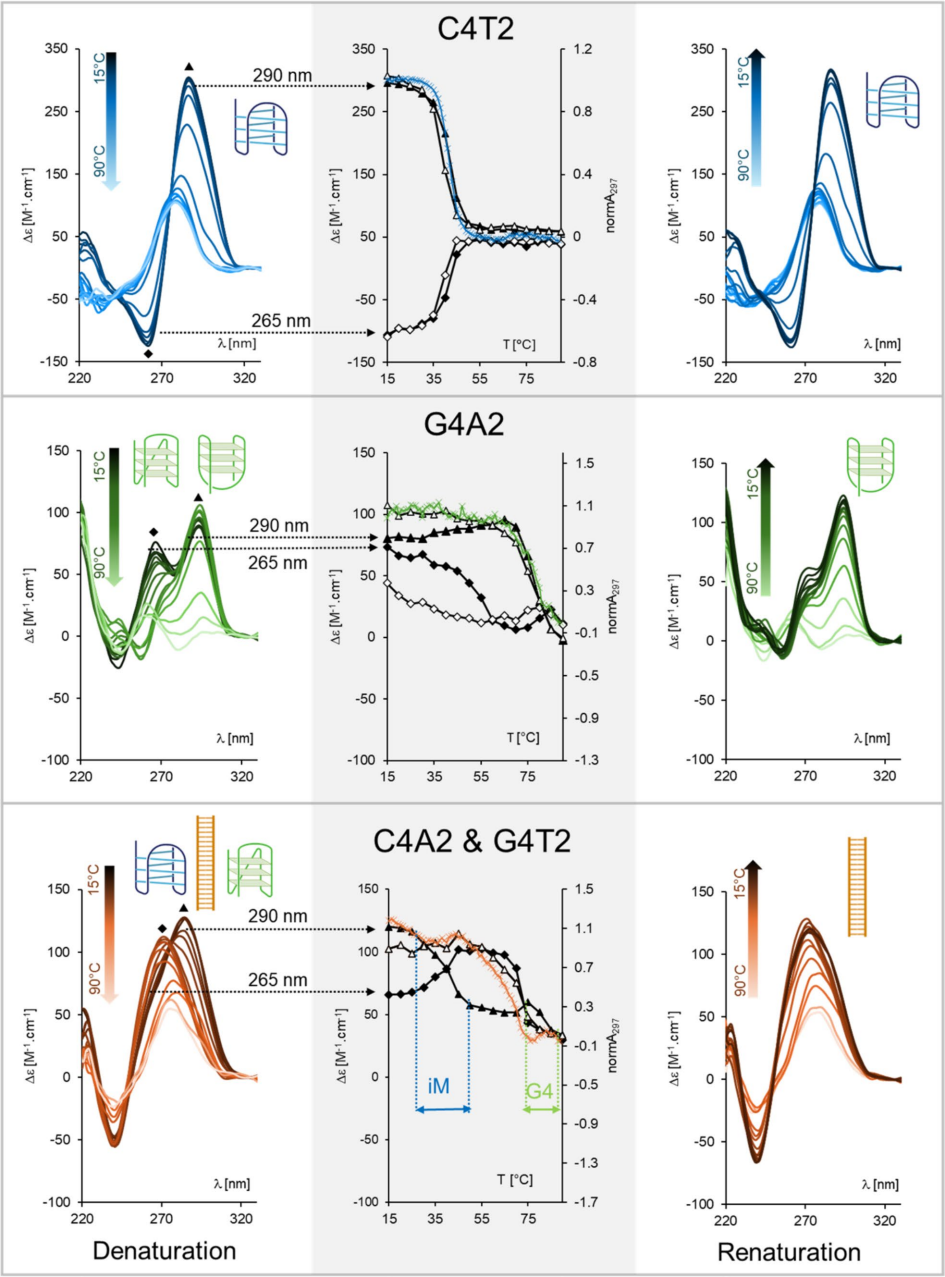
CD spectra and melting curves of C4T2 (upper row), G4A2 (middle row) and their mixture (bottom row) in 1 × IC buffer, pH 6 during thermal denaturation (left column) and renaturation (right column). Middle column shows melting curves observed by CD at indicated peaks, i.e. at 290 nm (triangles) or at 265 nm (diamonds) during the denaturation (full symbols) and renaturation (empty symbols) process. Normalized melting curves monitored by absorbance at 297 nm (for iM or G4) and 260 nm (for duplex) are shown (crosses in colors corresponding to particular structures). Inserts show schematic representations of structures involved

Based on the literature and our former results, we selected as a starting model sequences G4A2 and C4T2. Considering the primary sequence, C4T2 should form in 1 × IC buffer, pH 6 an i-motif with modest thermal stability (Školáková et al. 2019). For the complementary sequence G4A2 we expected in the same conditions formation of stable guanine quadruplex, but we cannot clearly predict its conformation. Thus, the two sequences should represent several DNA conformations with adequate stability. CD spectroscopic data confirmed our suggestions (Fig. 2, left column): a positive CD peak at 290 nm of C4T2 indicates the formation of iM (top) and two positive peaks of G4A2 at 265 and 290 nm indicate either a mixture of parallel and antiparallel G4, or a hybrid G4 arrangement (middle). Interestingly, upon temperature increase the CD spectrum of G4A2 first lost the positive peak at 265 nm at the expense of a small increase of the 290 nm peak around 55 °C. This spectral change might be attributed to a conformational change of G4 from parallel to antiparallel type. Then, the 290 nm peak disappeared as the G4 unfolded around 78 °C. The C4T2 iM melted through a simple two-state cooperative process at around 42 °C (Fig. 2, middle column).

When we mixed the two oligonucleotides, the C4T2 forming iM and the G4A2 forming G4, we observed the CD spectrum like the average of CD spectra of the two mixed structures (Fig. 2, bottom left). This indicates that the G4 and iM remained as separate species upon mixing, without the formation of the double helix. The spectrum is mainly characterized by a positive CD peak around 290 nm, where both the iM and antiparallel or hybrid G4 have positive CD. Interestingly, the positive CD peak characteristic of parallel G4 around 265 nm diminished due to the additive effect of the negative CD of iM at this wavelength. Upon increasing temperature, the positive CD maximum cooperatively shifted from 290 nm to around 270 nm. This effect is due to the melting of iM and simultaneous intervention of unfolded C-rich strand into folded G4. Increased temperature probably facilitates this intervention and helps G4 unwinding, so a regular double helix might form. The final spectrum is characteristic of GC-rich double helix of B-DNA type. Further increase in temperature caused helix denaturation, followed by a decrease of CD at 290 nm. These changes in CD, which were also partially followed by changes in absorption at 260 and 297 nm (Fig. 2, middle column), indicate sequential conformational changes in the solution due to temperature increase: (1) melting of iM of C4T2 (decrease in CD at 290 nm), around 35 °C, which is 7 °C lower than free iM; (2) formation of double helix (increase in CD at 265 nm), which must be necessarily accompanied by unfolding of G4; (3) existence of stable double helix between 45 and 65 °C (no changes in CD), thus we do not observe conformational change of G4 here, which free G4 undergoes around 55 °C. We hypothesize that the unfolded C-rich strand after iM denaturation binds G-strand with partially unfolded G4 during G4 transition to antiparallel form, which then might not form; (4) unfolding of double helix around 72 °C (decrease in CD at 265 nm), accompanied by slow formation of antiparallel G4 (small increase in CD at 290 nm); (5) denaturation of the G4 (decrease in CD at 290 nm) around 80 °C.

### DNA primary sequence variations modulate the equilibrium between canonical double helix and alternative structures

We were then interested in how the variations in the primary sequence of DNA influence individual transitions and especially the propensity to form alternative structures. For the sake of simplification, we studied only regular sequences, where each C or G tract within the sequence has the same length and, similarly, each loop region within the sequence has the same length. We studied sets of sequences with blocks of three or four C or G that allow the formation of alternative structures stable enough in our conditions and are still quite frequent within genomes. We also extended loop regions between 1 and 4 nucleotides to follow the most interesting variations of alternative structures in terms of conformation and thermodynamic stability. Finally, as the sequence of loop regions might influence the properties of alternative structures, we tested also sequences with flipped loop regions, i.e. T-loops in C-rich strand with A-loops in G-rich strand and the opposite case.

**Fig. 3:**
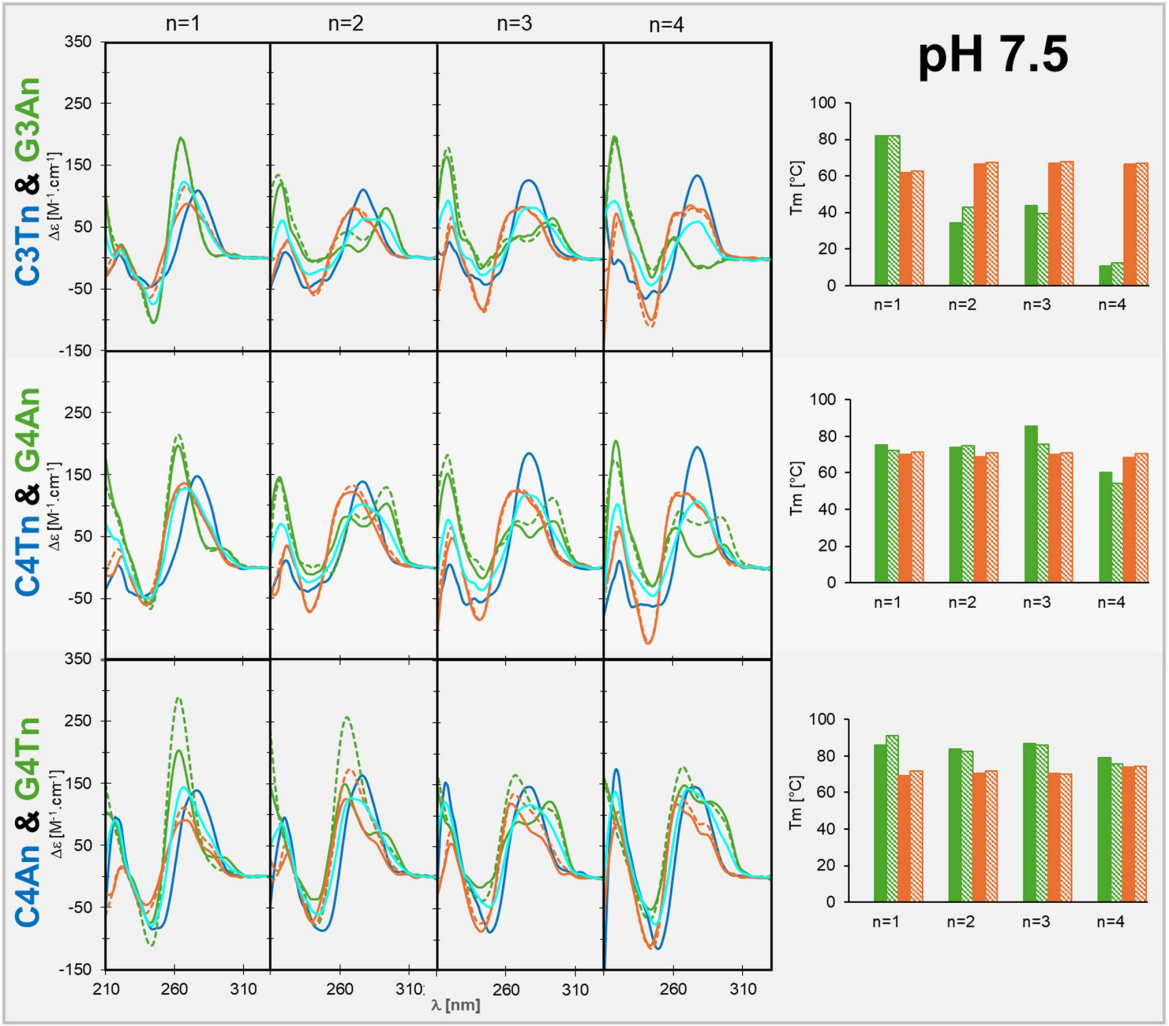
CD spectra (left panels) and melting temperatures (right) of C-rich DNA strands (blue), G-rich DNA strands (green) and their mixtures (orange) in 1 × IC buffer, pH 7.5. Solid lines and bars represent spectra before thermal denaturation and Tm, respectively. Dashed lines and bars represent spectra after thermal renaturation and Tren, respectively. The cyan CD spectra represent an average of the spectra of C-rich and G-rich strand

We started with C3Tn and complementary G3An (Table 1), following our previous studies (Školáková et al. 2019; Vorlickova et al. 2007) in intracellular buffer at close to physiological pH 7.5 (Fig. 3, upper row). None of the C3Tn strands for *n* = 1–4 form in this condition a significant portion of iM; a dominant positive CD peak is always at 277 nm with no transition to a longer wavelength. Tested eight-hour isothermal incubation at 23 °C after thermal denaturation does not support iM formation. Thus, no Tm was determined for C3Tn sequences in pH 7.5. Small variability in CD spectra between sequences might be due to an increased ratio of T in the sequence. In contrast, G3An strands might form guanine quadruplex: for *n* = 1 an extremely stable G4 of parallel type with Tm around 80 °C. Extending loops to two or three A (*n* = 2 or 3) leads to strong destabilization with Tm only around 40 °C and conformational change to mostly antiparallel G4, as shown by the shift of the maximum of positive CD from 264 to 290 nm (Fig. 3, upper row) and which is in line with literature (Hazel et al. 2004). For *n* = 2, but not for *n* = 3, an eight-hour incubation after denaturation slightly supports the formation of parallel G4, accompanied by an increase in overall Tren, compared to Tm. Preference for antiparallel G4 over more stable parallel ones thus results from kinetic aspects of G4 folding, as was shown for telomeric G4 (Boncina et al. 2012) and not from higher thermodynamic stability. For *n* = 4 no G4 formation was observed, and the CD corresponds to G/A rich unstructured single strand. Mixing of the two strands leads, for all loop lengths, to the formation of duplex DNA, as shown by a broad positive CD peak around 270 nm, which differs from the average CD spectra of C3Tn and G3An. The Tm of these duplexes is around 60 °C and does not significantly change with n and between Tm and Tren. Only for *n* = 1 after renaturation, the CD spectrum differs from that after mixing and it is very similar to the average CD spectrum of G and C strand mixture. This indicates that during renaturation a parallel G4 with higher thermal stability forms first and prevents Watson–Crick binding to the complementary C strand, which is unstructured in these conditions. Interestingly, such behavior was not observed after mixing the two strands without subsequent denaturation. We hypothesize that the G3A quadruplex, despite parallel CD, is not perfectly folded, but rather trapped in a mixture of kinetically preferred variants with sliding G-tract relative to each other. Such structures and namely their unpaired G would be susceptible to interaction with C in unstructured complementary strand, which might prevent transition to thermodynamically more stable, fully paired G4.

Next, we tried to increase the potential stability of alternative structures by increasing the number of G or C in respective tracts, resulting in C4Tn and G4An types of sequences (Table 1). Indeed, the C4Tn sequences, when introducing three- or four-T loops, formed a small fraction of iM, as indicated by redshift and increase in CD peak around 277 nm (Fig. 3, middle row). However, the iM stability is still too low to calculate Tm. Significant changes were observed in the thermodynamic stability of the complementary G4An strand: for *n* = 1 a parallel G4 forms, but with reduced Tm compared to G3A. This is in line with reported data from the Phan group (Devi et al. 2022) that short propeller loops, corresponding to a single nucleotide, cannot efficiently span more than three tetrads. Extension of loops leads to a conformational switch to an antiparallel G4, similarly as for three tetrad G3An sequences, but without a drop in melting temperature. The destabilizing effect of loop extension, observed for G3An sequences was compensated by the formation of an additional tetrad. Despite the higher stability of the G4 with longer loops, all the mixtures of C-rich and G-rich strands again quickly transform into DNA double helix. The increase in length of G and C tracts caused, besides increased stability of G4 and probably also of iM, an increase in stability of the duplex, with Tm around 70 °C for all *n*. Also, the increased loop length of the G4 facilitates interaction with nonstructured or only partially structured complementary strand and transition into the double helix (Kumar et al. 2008).

Finally, we tested the effect of purine/pyrimidine exchange in loops. The iM better accommodates pyrimidine loops and for example, T to A substitutions reduce iM thermal stability (Školáková et al. 2019). The same is true for parallel G4 where thymine propeller loops are thermodynamically preferred over adenine ones (Guedin et al. 2008). In contrast, antiparallel G4 might benefit from adenine loops, especially at the 3′ end, due to adenine stacking to a neighboring tetrad (Guédin et al. 2009). The switch in loop composition led to an overall increase in stability of G4 for all *n*, associated with higher content of parallel forms (Fig. 3, bottom row), and the Tm of G4T was even higher than that of G3A. The C-rich strand remained unstructured, though changes in CD were observed, namely a decrease of positive maximum around 277 nm. This might be attributed to the spectral influence of adenines, compared to thymines. After mixing with a complementary strand, the double helix was quickly formed, as shown by CD spectra, and no indices of a significant portion of G4 were observed, though the stability of the double helix did not increase upon loop sequence switch. The stabilization effect of G4 thus was overweighted by the stabilization of the double helix.

### Acidic pH, associated with cytosol of some cancer cells, drives the DNA conformational equilibrium slightly towards alternative structures

**Fig. 4:**
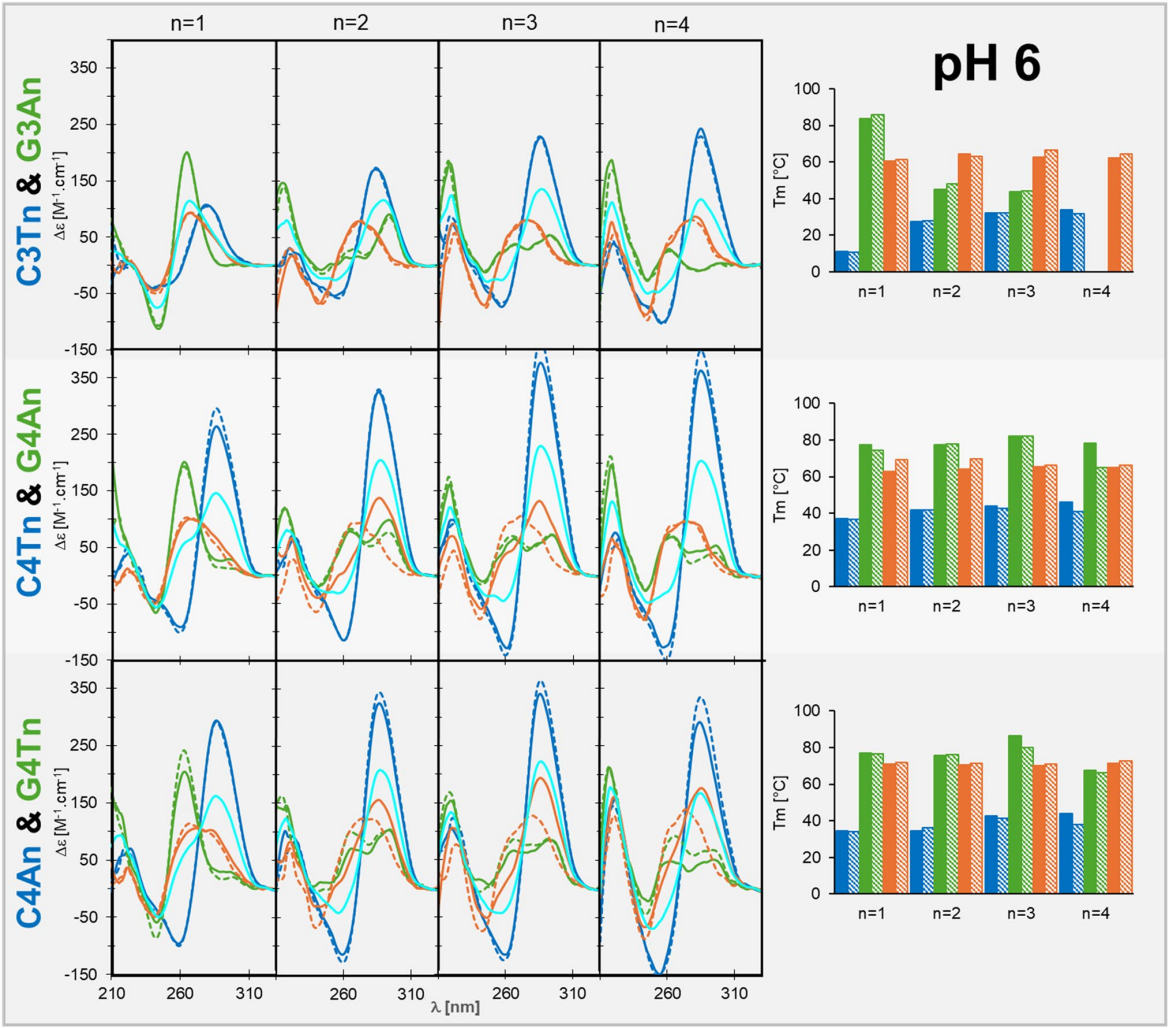
CD spectra (left panels) and melting temperatures (right) of C-rich DNA strands (blue), G-rich DNA strands (green) and their mixtures (orange) in 1 × IC buffer, pH 6. Solid lines and bars represent spectra before thermal denaturation and Tm, respectively. Dashed lines and bars represent spectra after thermal renaturation and Tren, respectively. The cyan CD spectra represent an average of the spectra of C-rich and G-rich strand

In the next part, we tested the effect of acidic pH, which should support the formation of iM and shift the conformation equilibria towards the presence of alternative structures (Phan and Mergny 2002). In contrast to previous reports, we used only pH 6, which might better mimic, for example, the acidification of cancer cells (Tafech and Stéphanou 2024). As expected, already for sequence C3T we observed the formation of iM (Fig. 4, upper row), though with limited stability due to the incorporation of several cytosines into loops. The optimal loop length for iM is three (Školáková et al. 2019) and for *n* = 3 or 4 we observed the highest Tm. Increasing iM ratio and stability are also reflected in CD spectra by redshift and increase of the maximum around 280–290 nm. The spectra and Tm of G4 in the complementary strand are similar to that in pH 7.5. Almost similar Tm in pH 6 and 7.5 were observed also for the double strand. The CD spectra upon mixing of G- and C-rich strands indicate the fast formation of the double helix; the presence of mildly stable iM thus does not alter the conformational equilibrium. No changes in the spectra of any structure were observed after thermal denaturation. This means that the tetramolecular structures unfold quickly in the presence of the complementary sequence and form a duplex, whose CD spectrum significantly differs from the average CD spectra of the mixed iM and G4. However, in the case of *n* = 1, the observed spectrum after mixing remains similar to the average of iM and G4 spectra. This indicates the presence of alternative structures. We observed indices of G4 for the same sequence in pH 7.5 after denaturation, and formation of iM in complementary strand should only support the presence of alternative structures at the expense of the double helix.

Extension of all C tracts by one C enhanced the stability of iM, as reflected by the increase in Tm to around 40 °C and height of the dominant positive CD peak (Fig. 4, middle and bottom row), with only a minor preference for T loops and longer loops, which is in line with detailed analysis of iM stability with respect to the primary sequence (Cheng et al. 2021). The stability and conformation of G4 formed by G4An or G4Tn sequences was not significantly affected by pH decrease. However, significant changes were observed for mixtures of C- and G-rich strands. For mixtures of C4Tn with G4An and *n* = 2 or 3, the CD spectrum of the mixture differs from the average of CD spectra of iM and G4, it does not resemble the spectrum of the double helix, it rather implies a complex mixture of forming double helix with residual fractions of iM and G4. The kinetics of transition from alternative structures to double helix is thus significantly slowed down by the formation of iM and within the measurement time, only part of the molecules undergoes conformation transition. After thermal denaturation, the CD spectra of all the mixtures belong to the B-form double helix. No such transition slowdown was observed for *n* = 1 and for *n* = 4, so there is no clear correlation between loop length and conformational transition properties. While the short loop penalizes iM, the long loop slightly destabilizes G4. In these cases the double helix forms quickly.

In the case of loop-reverted sequences, i.e. C4A4 with G4T4 mixtures (Fig. 4, bottom row), immediate formation of the double helix was observed only for *n* = 1. For *n* = 4 the conformation equilibrium is the most shifted towards alternative structures from all loop lengths tested and even after thermal denaturation there are still indices of iM presence in the CD spectra. The striking contrast in conformational transition between C4T4/G4A4 and C4A4/G4T4 variants is hard to explain, especially when the double helix is a bit more stable in the case of the latter one. One possible explanation might be in the transient formation of G:A pairs (Li et al. 1991) that could impede the proper formation of G4. However, no such behavior was observed in pH 7.5.

## Conclusions

In this work, we analyzed using a CD spectroscopy conformational equilibrium of a set of model G/C-rich DNA sequences with respect to the formation of alternative structures, namely cytosine i-motifs and guanine quadruplexes. CD spectroscopy was confirmed to be a suitable method for rapid and inexpensive conformational screening of nucleic acids, allowing us to test 144 samples, i.e. G- and C-rich complementary DNA chains and their mixture for 3 types of sequences with 4 different loop lengths at two different pHs before and after thermal denaturation. In most cases, after mixing complementary chains, a double helix is immediately formed, which remains the dominant form, regardless of subsequent denaturation and refolding. This means that the complementary strand can unfold an alternative structure, typically G4, to form a double helix, despite the high thermal stability of the G4, which indicates that the kinetic aspects of folding of particular structures take place. In specific cases, however, we have observed indices of stable alternative structures after chain mixing:

- C3T and G3A sequences in pH 6 remain separated after mixing and G3A still forms G4.
- In pH 7.5 the C3T and G3A sequences form a double helix immediately after mixing, but subsequent double helix denaturation might lead to the formation of G4.
- A mixture of C4An and G4Tn and loop-reversed C4Tn and G4An sequences with 3–4 nt loops assembles into a double helix rather slowly due to the presence of stable alternative structures.
- Lowering the pH, which might be associated with some pathological cellular states, leads to a decrease in the rate of the transition from alternative structures to double helical DNA.

**Fig. 5:**
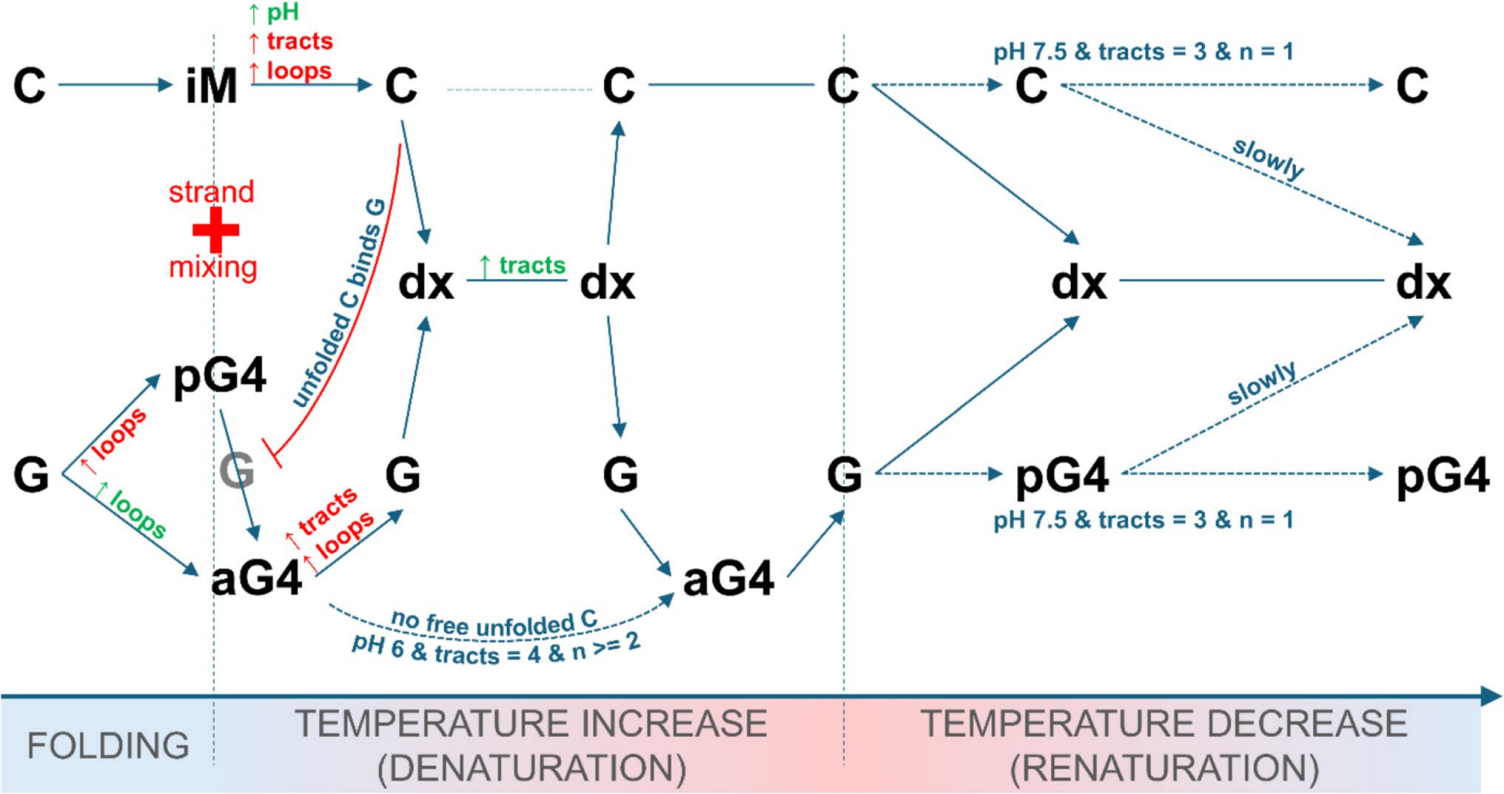
Schematic representation of transitions of nucleic acids structures, based on the data obtained with C4T2 and G4A2 in 1 × IC buffer, pH 6 (Fig. 2) as an example of the studied sequences, following iM and G4 formation and subsequent thermal denaturation and renaturation. C and G stand for unfolded C- and G-rich strands respectively, iM for cytosine i-motif, pG4 for dominantly parallel guanine quadruplex, aG4 for antiparallel quadruplex, tracts = length of individual C or G tracts, loops or *n* = length of individual loop regions within a sequence. The scheme is generalized for variations in primary sequence and conditions by colored notes. Green notes indicate positive (accelerating) effects on respective transitions, red notes indicate negative effects

In conclusion, G/C rich complementary DNA regions prone to the formation of alternative structures, frequently located in gene promoters, UTRs and other regulatory regions, prefer the formation of double-helical DNA over these alternative structures. However, specific primary sequences might obey this rule and form alternative structures that might be stable long enough to influence key nuclear processes. Observed conformational transitions are schematically depicted in Fig. 5.

Moreover, all the data presented here were observed on regular sequences possibly able to incorporate all C into C:C^+^ pairs in iM core or all G into guanine tetrads in G4 core. However, the dominant portion of native sequences in genomes does not follow such regular arrangement of C and G tracts. Prediction of conformational equilibria in these irregular sequences might significantly differ from regular sequences as there might be additional effects, for example, the G4 entropic stabilization due to surplus Gs, forming G-registers (Harkness and Mittermaier 2016), G4 rescue due to spare tire effects (Dvořáková et al. 2017; Fleming et al. 2015) and many other. For all such studies, however, CD spectroscopy might be still an ideal starting point.

## Data Availability

All data presented in the article are available upon request from the authors.
